# Multimodal contrastive learning for integrating molecular representations and cellular phenotypes in drug-target interaction prediction

**DOI:** 10.1093/bioinformatics/btag368

**Published:** 2026-08-21

**Authors:** Ying-Ju Lai, Tianyuzhou Liang, Po-Yuan Chen, Yu-Che Tsai, George C Tseng, Yufei Huang, Yu-Chiao Chiu

**Affiliations:** UPMC Hillman Cancer Center, University of Pittsburgh School of Medicine, Pittsburgh, PA 15232, United States; Department of Biostatistics and Health Data Science, University of Pittsburgh, Pittsburgh, PA 15261, United States; UPMC Hillman Cancer Center, University of Pittsburgh School of Medicine, Pittsburgh, PA 15232, United States; Integrative Systems Biology Program, University of Pittsburgh School of Medicine, Pittsburgh, PA 15232, United States; Institute of Biomedical Sciences, Academia Sinica, Taipei 115201, Taiwan; Department of Computer Science and Information Engineering, National Taiwan University, Taipei 106319, Taiwan; Department of Biostatistics and Health Data Science, University of Pittsburgh, Pittsburgh, PA 15261, United States; UPMC Hillman Cancer Center, University of Pittsburgh School of Medicine, Pittsburgh, PA 15232, United States; Department of Medicine, University of Pittsburgh School of Medicine, Pittsburgh, PA 15232, United States; UPMC Hillman Cancer Center, University of Pittsburgh School of Medicine, Pittsburgh, PA 15232, United States; Department of Biostatistics and Health Data Science, University of Pittsburgh, Pittsburgh, PA 15261, United States; Integrative Systems Biology Program, University of Pittsburgh School of Medicine, Pittsburgh, PA 15232, United States; Department of Medicine, University of Pittsburgh School of Medicine, Pittsburgh, PA 15232, United States

## Abstract

**Motivation:**

Accurate prediction of drug-target interactions (DTIs) is fundamental to drug discovery and mechanistic understanding. While deep learning has advanced computational DTI prediction, most existing methods rely primarily on molecular structural representations, including drug structures and protein sequences, while overlooking cellular phenotypes that reflect downstream biological effects. Cell Painting enables high-content morphological profiling that captures systems-level responses to chemical and genetic perturbations but remains underutilized in DTI modeling. Integrating molecular information with cellular phenotypes offers an opportunity to improve both predictive performance and biological interpretability.

**Results:**

We propose a two-stage contrastive learning framework integrating drug structures, protein sequences, and Cell Painting morphological profiles into a unified embedding space. Stage 1 learns modality-specific representations independently from structure-based and image-based data; Stage 2 aligns these via multi-positive contrastive learning to bridge molecular structural information with cellular phenotypes. Cross-modal retrieval achieves median Recall@10 values of 0.77 (random split) and 0.33 (scaffold split), outperforming bilinear and random baselines. In external DTI prediction on the BIOSNAP dataset, our model achieves an AUC of 0.92 with image-based representations and 0.90 under structure-only settings, surpassing existing methods. Model interpretation via integrated gradients reveals pathway-specific morphological signatures associated with drug targets, providing biologically interpretable insights into drug mechanisms.

**Availability:**

https://github.com/YJRubyLai/Unified-DTI

## 1 Introduction

Studying DTI is foundational for the identification of therapeutic targets, repurposing of existing drugs, and elucidation of molecular mechanisms of action (Sneader 2005). Experimental identification and validation of DTIs are time-consuming and resource-intensive, highlighting the need for computational methods that can efficiently prioritize candidate interactions. Recent advances in deep learning have produced numerous computational DTI prediction methods (Iqbal *et al.* 2024). However, most existing approaches depend primarily on molecular structural representations of drugs and target proteins, overlooking rich cellular and phenotypic contexts that could improve prediction performance and biological interpretability.

Beyond drug molecular structures and protein sequences, chemical and genetic perturbations induce cellular phenotypic responses that reflect downstream biological processes. Cell Painting is a high-throughput imaging assay that captures these responses through multiplexed fluorescence microscopy and generates high-content morphological profiles encoding systems-level functional effects across diverse cellular compartments (Bray *et al.* 2016, Moshkov *et al.* 2024). These features have been widely applied to mechanism-of-action analysis, toxicity prediction, and gene-compound functional mapping (Iyer *et al.* 2024, Seal *et al.* 2025). Importantly, Cell Painting reveals biological effects not apparent from molecular features alone, providing complementary insight into drug and target activity (Bhate *et al.* 2026). However, such phenotypic data remain largely underutilized in DTI prediction. Integrating molecular structural information with cellular phenotypes remains challenging because these modalities operate at different biological scales: structure-based representations capture biochemical interactions between drugs and proteins, whereas phenotypic profiles reflect downstream cellular and functional consequences. Bridging these complementary data sources through unified representation learning could improve generalization to unseen compounds and targets, enabling a more comprehensive and mechanistically informed characterization of drug-target relationships.

Contrastive learning has proven effective for aligning heterogeneous modalities. Methods such as Contrastive Language-Image Pretraining (CLIP) demonstrate that contrastive objectives can learn shared embedding spaces by bringing matched pairs closer while separating mismatched pairs, enabling cross-modal retrieval and transfer learning (Radford *et al.* 2021, Gao *et al.* 2023). This paradigm has been successfully applied in biological contexts, including protein-ligand binding prediction and drug-disease association (Li *et al.* 2022, Gao *et al.* 2023). These approaches capture biologically meaningful relationships across diverse data types without explicit feature engineering, making contrastive learning a natural choice for integrating molecular and phenotypic information in DTI modeling.

Inspired by these advances, we propose a two-stage contrastive learning framework that learns modality-specific DTI representations from molecular representations and cellular images and aligns them into a unified multimodal embedding space. To our knowledge, this is the first framework to explicitly bridge molecular representations with high-content cellular phenotypes through contrastive alignment. The resulting space enables cross-modal retrieval, robust DTI prediction, and interpretable feature attribution, jointly modeling biochemical events and their downstream functional effects.

## 2 Methods

**Figure 1:**
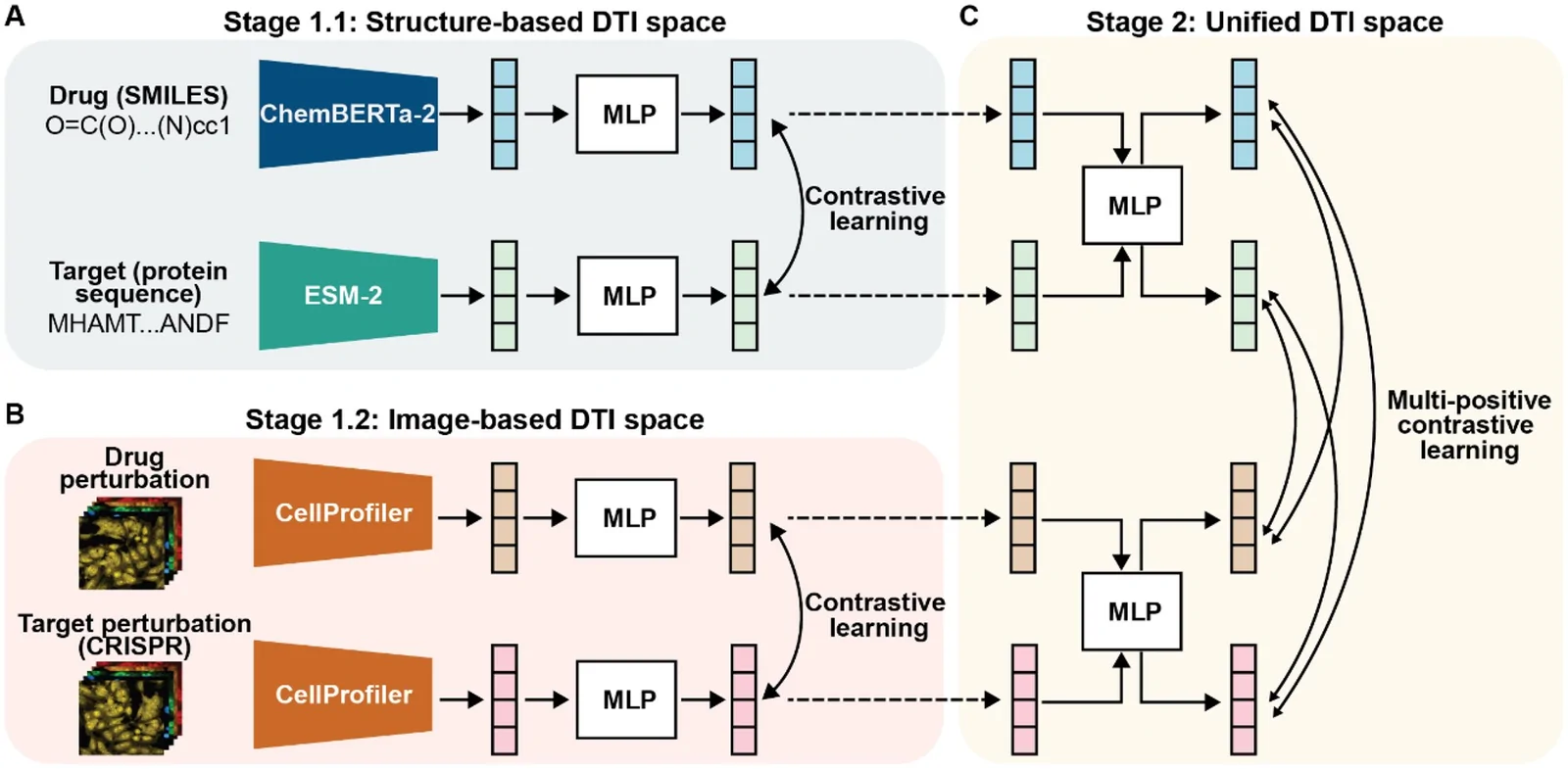
Overview of the proposed two-stage contrastive learning framework for unified multimodal DTI representation. (A) Stage 1.1: Structure-based DTI space from SMILES (ChemBERTa-2) and protein sequences (ESM-2) (B) Stage 1.2: Image-based DTI space from drug and CRISPR Cell Painting profiles (C) Stage 2: Unified DTI space learned via multi-positive contrastive learning.

As illustrated in Fig. 1, the proposed framework consists of two sequential stages designed to construct a unified multimodal embedding space for DTIs. In Stage 1, modality-specific representations are learned independently via contrastive learning: a molecular structure-based DTI space aligning drug representations encoded from SMILES strings using ChemBERTa-2 (Ahmad *et al.* 2022) with protein representations encoded from amino acid sequences using ESM-2 (Lin *et al.* 2023), and an image-based DTI space aligning Cell Painting morphological profiles of chemical and genetic perturbations. In Stage 2, these modality-specific embeddings are integrated through multi-positive contrastive learning to bridge structural information with cellular phenotypic responses within a shared latent space. The resulting unified representations are evaluated on cross-modal DTI retrieval, robust DTI prediction, and feature importance analyses.

### 2.1 Data curation and preprocessing

#### 2.1.1 DTI annotations for model training and validation

To construct a unified DTI representation space, we curated 282 236 DTI pairs from the MOTIVE dataset (Arevalo *et al.* 2024), which aggregates compound-gene associations from multiple biomedical databases including DGIdb and OpenBioLink. We retained only direct binding interaction data between drugs and protein targets, excluding indirect or associative relationships. This filtering ensures that the model is trained on high-confidence, physically grounded interactions suitable for multimodal representation learning. Following the MOTIVE evaluation strategy, we adopted both cold-drug and cold-target splits to evaluate model generalization to unseen drugs and targets.

To evaluate the predictive utility and generalizability of the learned unified embeddings, we used the BIOSNAP dataset as an external benchmark, which comprises labeled interacting and non-interacting drug-target pairs (Leskovec and Sosič 2016). To ensure compatibility with our multimodal framework, we retained only pairs with corresponding Cell Painting profiles. The final evaluation set comprised 1084 DTI pairs, including 776 interacting and 308 non-interacting pairs. The selected subset was confirmed by silhouette analysis to broadly cover the embedding-space distribution of the full BIOSNAP dataset, with silhouette scores near zero indicating no clear separation in the learned multimodal representation space.

#### 2.1.2 Molecular representations of drugs and proteins

For structural drug representations, SMILES strings were retrieved from DrugBank, PubChem, and ChEMBL (Gaulton *et al.* 2012, Knox *et al.* 2024, Kim *et al.* 2025). All SMILES strings were canonicalized using RDKit to standardize molecular encodings and eliminate redundancy (Bento *et al.* 2020). For protein representations, amino acid sequences were restricted to human proteins sourced from NCBI and UniProt (Pruitt *et al.* 2012, UniProt Consortium 2019). To maintain computational efficiency, sequences exceeding 2048 amino acids were excluded, representing approximately 1.4% of the dataset. After preprocessing and alignment with curated DTI pairs, the final dataset comprised 72 356 unique SMILES strings and 8252 unique protein sequences.

#### 2.1.3 Image-based phenotypic profiles (Cell Painting)

Image-based representations were sourced from the JUMP Cell Painting Gallery (cpg0016) (Chandrasekaran *et al.* 2023). This repository provides 116 753 chemical perturbations and 7977 CRISPR-mediated gene knockouts, enabling the mapping of both drugs and targets into a high-dimensional morphological space.

### 2.2 Feature representation and encoding

#### 2.2.1 Drug structure encoder

ChemBERTa-2 is a transformer-based chemical language model that learns contextualized molecular representations from SMILES strings (Ahmad *et al.* 2022). We used the ChemBERTa-77M-MTR model, which was pretrained with a multi-task regression objective on 77 million unique SMILES strings from PubChem. Canonicalized SMILES were encoded using this model, and 384-dimensional molecular embeddings were extracted from the final layer [CLS] token.

#### 2.2.2 Protein sequence encoder

ESM-2 is a transformer-based protein language model that learns contextualized protein representations from amino acid sequences (Lin *et al.* 2023). We used the ESM-2 model (t33_650M_UR50D), a 33-layer architecture with 650 million parameters pretrained on the UniRef50 with masked language modeling. Protein sequences were encoded using this model, and 1280-dimensional protein embeddings were extracted from the final encoder layer [CLS] token.

#### 2.2.3 Cell Painting feature extraction

Cell Painting images were processed using CellProfiler, an open-source platform for quantitative analysis of high-content microscopy data (Stirling *et al.* 2021). Cells were segmented and thousands of morphological features describing shape, intensity, texture and spatial organization were extracted across channels. Extracted features were preprocessed as previously described (Stirling *et al.* 2021), including removal of low-variance features, normalization, batch correction and feature selection. After preprocessing, chemical perturbations were represented by 737 features, while CRISPR gene knockout perturbations were represented by 599 features. These profiles were used as image-based cellular representations for downstream modeling.

### 2.3 Stage 1: structure- and image-specific DTI spaces

#### 2.3.1 Structure-based DTI space

To learn aligned drug-target representations, we employed a symmetric contrastive framework that jointly optimizes drug-to-target and target-to-drug associations within a shared latent space.

Given a batch of N known DTI pairs $\{(d_{i},t_{i}){\}}_{i=1}^{N}$, each drug $d_{i}$ is encoded from its SMILES string using ChemBERTa-2, and each target $t_{i}$ is encoded from its amino acid sequence using ESM-2. The resulting embeddings are projected into a k-dimensional structure-based DTI space using modality-specific multilayer perceptron (MLP) projection heads, yielding drug embeddings $z_{i}^{\left(d\right)}\in R^{k}$ and target embeddings $z_{i}^{\left(t\right)}\in R^{k}$.

A symmetric contrastive objective is employed to maximize similarity between known DTI pairs while minimizing similarity to non-interacting pairs within the same batch. For each drug embedding $z_{i}^{\left(d\right)}$, its corresponding target embedding $z_{i}^{\left(t\right)}$ is treated as a positive sample, while all other target embeddings ${\left\{z_{j}^{\left(t\right)}\right\}}_{j\neq i}$ serve as negatives. The drug-to-target contrastive loss is defined as:


$$L_{d\to t}=-\frac{1}{N}\sum_{i=1}^{N}\log\frac{\exp(\mathrm{sim}(z_{i}^{\left(d\right)},z_{i}^{\left(t\right)})/\tau )}{\sum_{j=1}^{N}\exp(s\mathrm{im}(z_{i}^{\left(d\right)},z_{j}^{\left(t\right)})/\tau )},\;$$

where sim(⋅,⋅) denotes cosine similarity and τ is a temperature hyperparameter. Analogously, a target-to-drug contrastive loss is defined by treating each target embedding as the anchor and its paired drug embedding as the positive:


$$L_{t\to d}=-\frac{1}{N}\sum_{i=1}^{N}\log\frac{\exp(\mathrm{sim}(z_{i}^{\left(t\right)},z_{i}^{\left(d\right)})/\tau )}{\sum_{j=1}^{N}\exp(s\mathrm{im}(z_{i}^{\left(t\right)},z_{j}^{\left(d\right)})/\tau )}\;$$

The final structure-based contrastive objective is computed as the average of the two directional losses:


$$L_{\text{struct}}=\frac{1}{2}\left(L_{d\to t}+L_{t\to d}\right)$$

This symmetric formulation encourages mutual alignment between drug and target representations in the learned structure-based DTI embedding space.

#### 2.3.2 Image-based DTI space

To learn the image-based DTI space, we applied the same symmetric contrastive framework, replacing SMILES and protein sequences with Cell Painting morphological profiles. CellProfiler (Stirling *et al.* 2021) is an open-source image analysis software that extracts interpretable quantitative morphological features from high-throughput microscopy images. Drug- and target-induced perturbation features extracted by CellProfiler were projected into a k-dimensional embedding space using modality-specific MLP heads. The resulting embeddings were optimized with the same bidirectional contrastive loss to align interacting pairs while separating non-interacting pairs within each batch.

### 2.4 Stage 2: unified DTI space

In Stage 2, representations from Stage 1 were projected into a unified multimodal latent space via modality-specific MLP heads. A symmetric contrastive framework was employed with two alignment objectives: (1) intra-entity consistency between structure- and image-based representations of the same drug or target, and (2) cross-entity alignment preserving DTIs across modalities. For each known DTI pair, four cross-modal associations were treated as positives: drug structure-drug image, target structure-target image, drug structure-target image, and drug image-target structure. This many-to-many matching strategy ensures that the learned unified space captures both entity-level consistency and interaction-level associations.

Given the many-to-many relationships, we adopted a multi-positive InfoNCE objective. For each anchor i, let P(i) denote the set of all samples sharing the same label. The directional loss is:


$$L_{\mathrm{str}\to \mathrm{img}}=-\frac{1}{N}\;\sum_{i=1}^{N}\frac{1}{\left||P(i)|\right|}\sum_{\;p\epsilon P(i)}\log\frac{\exp(\mathrm{sim}(z_{i}^{\left(\mathrm{str}\right)},z_{p}^{\left(\mathrm{img}\right)})/\tau )}{\;\sum_{j=1}^{N}\exp(s\mathrm{im}(z_{i}^{\left(\mathrm{str}\right)},z_{j}^{\left(\mathrm{img}\right)})/\tau )\;}$$

with symmetric loss $L=(L_{\mathrm{str}\to \mathrm{img}}+L_{\mathrm{img}\to \mathrm{str}})/2$, where τ is the temperature, and $z_{i}^{\left(\mathrm{str}\right)}$, $z_{i}^{\left(\mathrm{img}\right)}$ denote the L2-normalized projections of sample i from each modality.

Unlike standard InfoNCE, this formulation averages the log-likelihood over all positives in P(i), encouraging each anchor to align with all matched counterparts in the unified space.

### 2.5 Downstream tasks and evaluation

#### 2.5.1 Intra- and cross-entity retrieval

To evaluate the learned unified space, we performed cross-modal retrieval tasks reflecting practical drug discovery scenarios such as target identification and drug prioritization. Given a query from one modality (drug structure, drug image, protein sequence, or target image), the task retrieves the corresponding entity or interacting counterpart from another modality ranked by cosine similarity.

We benchmarked on 1000 DTI pairs from the held-out test set, evaluating both intra-entity alignment (drug structure ↔ drug image; target structure ↔ target image) and cross-entity interaction retrieval (drug structure ↔ target structure, drug structure ↔ target image, drug image ↔ target structure). We compared our unified model with two baselines. The first is a bilinear contrastive model, where similarity between two embeddings is computed using a learned bilinear form: $s_{\mathrm{ij}}=x_{i}^{⊤}Wy_{j},\;$with W a trainable weight matrix optimized under a symmetric contrastive objective. The second is a random retrieval baseline representing chance-level performance.

Retrieval performance was assessed using Recall@*K* (*K *= 1, 10), which measures the proportion of queries for which the true match appears among the top-*K* candidates. Formally, a set of N queries, Recall@*K* is calculated as:


$$\mathrm{Recall}@K=\frac{\;1\;}{\;N\;}\sum_{i=1}^{N}1({\mathrm{rank}}_{i}\leq K),\;$$

where ${\mathrm{rank}}_{i}$ denotes the ranking position of the true match for query i, and 1(⋅) is the indicator function.

#### 2.5.2 DTI prediction

To evaluate the predictive utility of the unified embeddings, we performed a downstream DTI prediction task. Drug and target representations were extracted from the unified multimodal space under different feature configurations, including structure-only and image-enhanced settings. These embeddings were averaged to construct pairwise interaction features for each DTI instance. A random forest classifier was then trained on these features to predict binary interaction labels, enabling direct assessment of how well the learned representations capture drug-target associations. We also evaluated Cell Painting features using DeepProfiler (Moshkov *et al.* 2024), a deep learning model for extracting features from Cell Painting images of chemical and genetic perturbations, and applied the same prediction pipeline for comparison.

For comparison with existing DTI prediction methods, we benchmarked against MIF-DTI (Shan *et al.* 2025) and MCANet (Li *et al.* 2022) under a structure-only configuration. MIF-DTI integrates SMILES strings, amino acid sequences, and graph-based structural features, through a fusion module for DTI prediction. MCANet predicts DTIs using a multi-head cross-attention mechanism to model interactions between drug SMILES and protein sequence representations. Model performance was evaluated using 5-fold cross-validation, reporting the mean and standard deviation of AUC, accuracy (ACC), and F1 score.

#### 2.5.3 Integrated gradient analysis

To examine the role of morphological features in the unified model, we employed Integrated Gradients (IG) to estimate feature-level contributions from Cell Painting profiles. IG is an attribution method that quantifies the influence of each input feature on the model output by accumulating gradients along a linear interpolation path between a baseline reference and the input sample (Sundararajan *et al.* 2017).

For a model F(x) and an input vector $x\in R^{d}$, the attribution for feature *k* is computed as:


$${\mathrm{IG}}_{k}(x)=(x_{k}-x_{k}^{\prime })\int_{0}^{1}\frac{\partial F(x^{\prime }+\alpha (x-x^{\prime }))}{\partial x_{k}}\;d\alpha ,\;$$

where $x^{\prime }$ denotes the baseline, and controls interpolation between baseline and input. We applied IG to the trained model to identify key CellProfiler-derived morphological features under both chemical and CRISPR perturbations, highlighting biologically meaningful descriptors that contribute to the learned DTI representations.

### 2.6 Model training and implementation details

The two-stage framework was trained and evaluated under both random and scaffold splits. Random splitting permits structurally similar compounds to appear in both training and test sets, whereas scaffold splitting partitions molecules by core chemical frameworks to ensure that test scaffolds remain unseen during training (Wu *et al.* 2018). The latter prevents the model from exploiting structural familiarity, requiring genuine generalization to novel compounds and providing a more stringent and realistic evaluation of generalization in drug discovery.

Training was conducted in stages with modality-specific datasets. Stage 1.1 (structure-based DTI) used 116 267 training pairs and 29 067 test positive pairs. Stage 1.2 (image-based DTI) included 17 999 training and 4000 test positive pairs due to limited Cell Painting availability. Stage 2 (Unified DTI) used 15 292 training and 5 746 test positive pairs.

In all contrastive stages, negative samples were implicitly defined as in-batch negatives.

All experiments were performed on NVIDIA A100 GPUs (40 GB) using the Adam optimizer with learning rate 1 × 10^−4^, batch size 256, and contrastive temperature τ = 0.07.

## 3 Results

### 3.1 Overview of Stage 1 and Stage 2 DTI spaces

**Figure 2:**
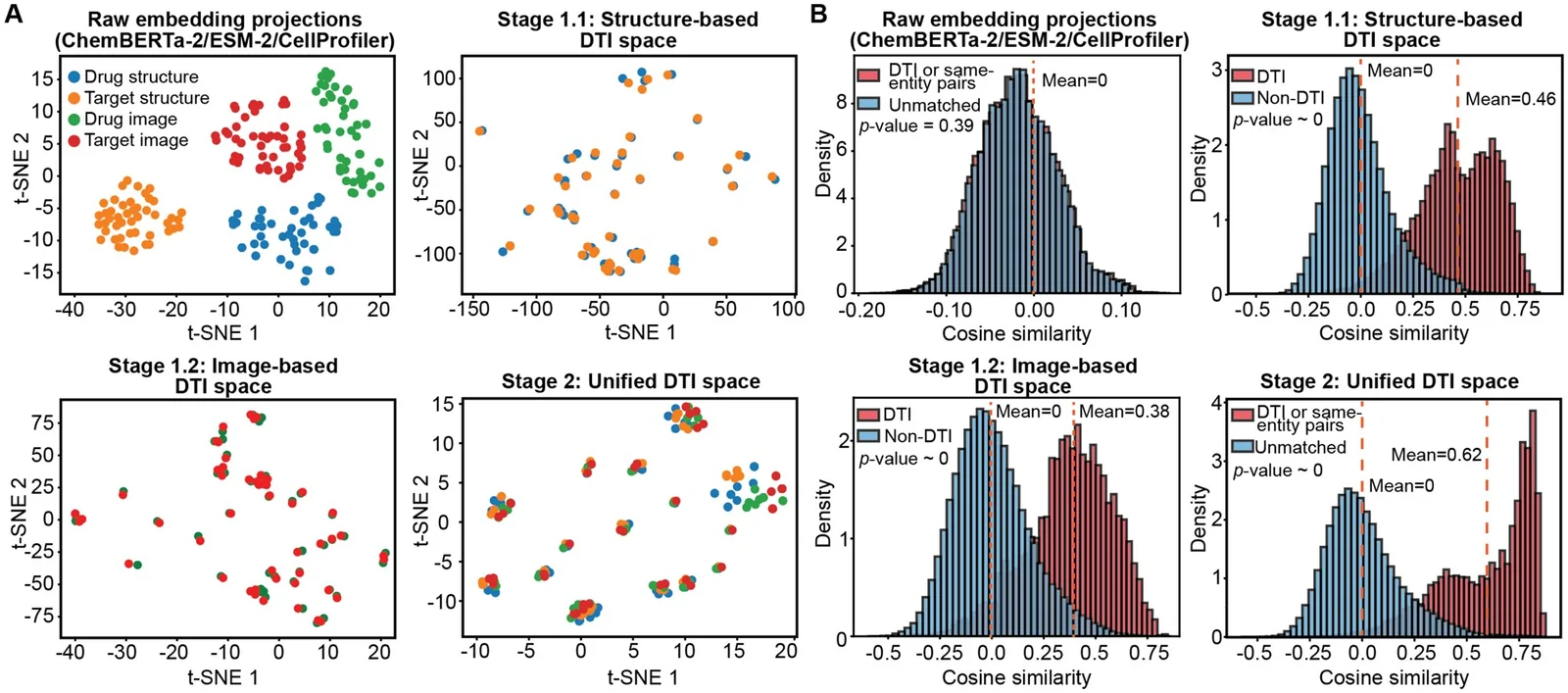
Visualization of embedding spaces and similarity distributions across training stages. (A) t-SNE plots of a random subset of 50 DTI pairs to illustrate grouping patterns in the raw and contrastive learning-based embedding spaces. Raw embeddings from ChemBERTa-2 and ESM-2 are projected into 512-dimensional spaces to enable comparison. (B) Histograms of cosine similarity comparing positive pairs (DTI pairs, or same drug-drug or target-target pairs) with negative unmatched samples. The *P*value comparing positive and negative pairs were calculated using the Mann-Whitney U test.

To assess progressive alignment in our two-stage contrastive learning framework, we visualized embedding spaces at each stage using t-SNE on 50 randomly sampled DTI pairs from the test set. Cosine similarity distributions for matched pairs (DTIs or intra-entity correspondences) and unmatched pairs were examined to assess separation in the embedding space (Fig. 2).

Before contrastive learning, modality-specific representations were clearly separated with no discriminative capability, as cosine similarities for matched and unmatched pairs were nearly identical and mean values close to zero. After Stage 1, alignment improved within each modality. In the structure-based space, interacting drug-target pairs were drawn closer together, reflecting recognition and binding relationships. Interacting pairs showed substantially higher cosine similarity (0.46 ± 0.19) than non-interacting pairs (0.00 ± 0.16), with a significant difference between groups (Mann-Whitney test, *P* value = 0).

Similarly, in the image-based space, drug- and target-induced morphological embeddings exhibited closer alignment, consistent with interaction-associated phenotypic responses. Interacting pairs showed higher cosine similarity (0.38 ± 0.20) than non-interacting pairs (0.003 ± 0.18).

In the Stage 2 unified space, structure- and image-based embeddings were jointly aligned. Representations of the same drug or target clustered together, and interacting drug-target pairs were also positioned nearby, indicating integration of molecular structural properties and phenotypic effects. The unified space showed the strongest separation, with higher cosine similarity for matched pairs (0.62 ± 0.23) than for unmatched pairs (0.008 ± 0.19).

Consistent with these findings, under the more stringent MOTIVE cold-source and cold-target splits evaluating generalization to unseen drugs or targets, the model still maintained clear separation between interacting and non-interacting pairs. In the cold-source split, cosine similarity increased from 0.04 vs. 0.04 before training to 0.40 vs. 0.09 in the Stage 2 unified space, while the cold-target split showed similar separation (0.35 vs. 0.03). These results demonstrate that the two-stage framework progressively integrates structural and morphological information into a biologically coherent DTI representation space.

### 3.2 Retrieval performance

**Figure 3:**
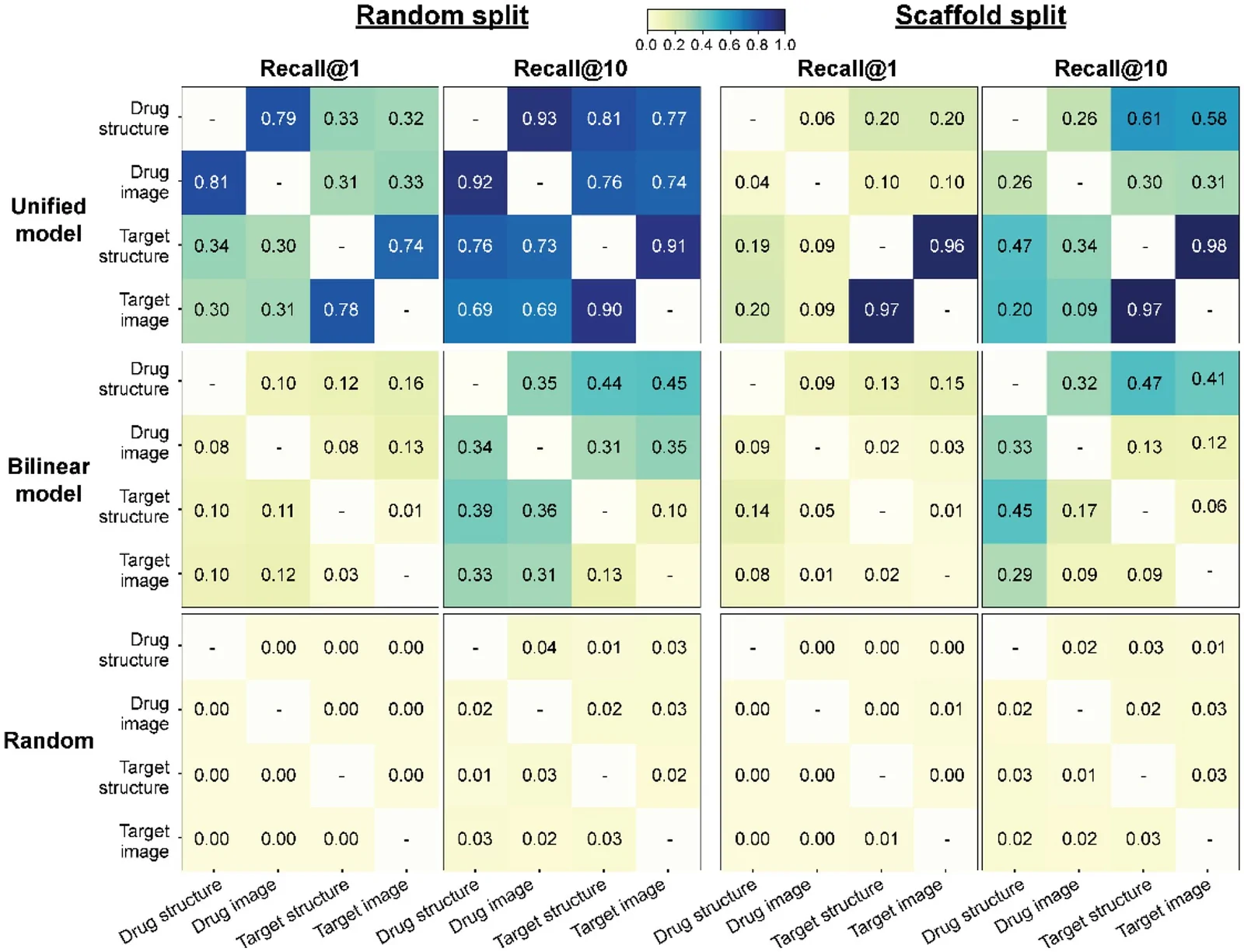
Cross-modal retrieval performance across drug and target structure/image modalities under random and scaffold splits. Recall@1 and Recall@10 are shown for the unified model, bilinear model, and random baseline.

Cross-modal retrieval performance across all modalities is summarized in Fig. 3. Under random splitting, our unified model achieved strong performance across both intra-entity and cross-entity retrieval tasks (Fig. 3A). Intra-entity alignment was particularly robust, with drug structure → drug image and target structure → target image achieving Recall@1 values of 0.79 and 0.78, respectively, and exceeding 0.90 at Recall@10. Cross-entity retrieval also remained substantial, with Recall@10 values ranging from 0.69 to 0.81, indicating that interacting drug-target pairs are effectively captured across heterogeneous modalities.

Under the more stringent scaffold split, retrieval performance decreased but remained meaningful. Intra-target alignment remained particularly strong (Fig. 3B), with target structure → target image achieving Recall@1 of 0.96 and Recall@10 of 0.98. Cross-entity retrieval also retained meaningful performance, with drug structure → target structure and drug structure → target image reaching Recall@10 values of 0.61 and 0.58, respectively, demonstrating generalization to structurally novel compounds.

In contrast, the bilinear baseline performed substantially worse under both splits, with most Recall@1 values below 0.16 and Recall@10 generally below 0.47, while random retrieval yielded near-zero performance, confirming that the improvements of our model reflect genuine cross-modal alignment rather than chance. Together, these results show that the unified embedding space captures meaningful drug-target relationships.

### 3.3 DTI prediction

To evaluate the predictive utility of the learned representations, Table 1 summarizes DTI prediction performance on the BIOSNAP dataset across different combinations of structure- and image-based representations. Incorporating image-based information improved performance. Image-derived embeddings for both drugs and targets achieved the best overall results (AUC = 0.92, ACC = 0.84, F1 = 0.89). Mixed-representation settings also performed competitively; notably, combining image-based drug embeddings with structure-based target embeddings yielded an AUC of 0.91 and the highest F1 score (0.90), suggesting complementary information between phenotypic drug responses and protein sequences. In contrast, combining drug structure with target image embeddings yielded the lowest performance, indicating that target morphology alone may be less informative when combined with drug structural features.

**Table 1:** DTI prediction performance.

Drug	Target	AUC	ACC	F1
**Our model**				
Structure	Structure	0.8967 (0.0198)	**0.8967 (0.0198)**	0.8826 (0.0167)
Structure	Image	0.8719 (0.0154)	0.7998 (0.0180)	0.8624 (0.0137)
Image	Structure	0.9131 (0.0167)	0.8524 (0.0193)	**0.8965 (0.0154)**
Image	Image	**0.9185 (0.0179)**	0.8441 (0.0197)	0.8881 (0.0141)
**MIF-DTI**				
Structure	Structure	0.8395 (0.0005)	0.8000 (0.0004)	0.8707 (0.0169)
**MCANet**				
Structure	Structure	0.8520 (0.0001)	0.7904 (0.0003)	0.8544 (0.0162)
**DeepProfiler**				
Image	Image	0.7997 (0.0265)	0.7730 (0.0157)	0.8491 (0.0117)

Results are reported as mean (standard deviation) over 5-fold cross-validation. The best performance in each column is shown in bold.

Compared with existing structure-based methods, our structure-only configuration achieved an AUC of 0.90, outperforming MIF-DTI (0.84) and MCANet (0.85), indicating that the proposed contrastive learning framework alone learns more expressive and transferable DTI representations than prior approaches. Incorporating Cell Painting-derived phenotypic representations further improved performance, with image-enhanced configurations achieving the best overall results (AUC up to 0.92), demonstrating that cellular phenotypes provide complementary biological information beyond molecular representations alone. In contrast, an alternative image-based representation using DeepProfiler achieved a lower AUC of 0.80. Overall, these results demonstrate that the unified embeddings provide strong predictive power through the complementary integration of molecular and phenotypic information.

### 3.4 IG analysis

**Figure 4:**
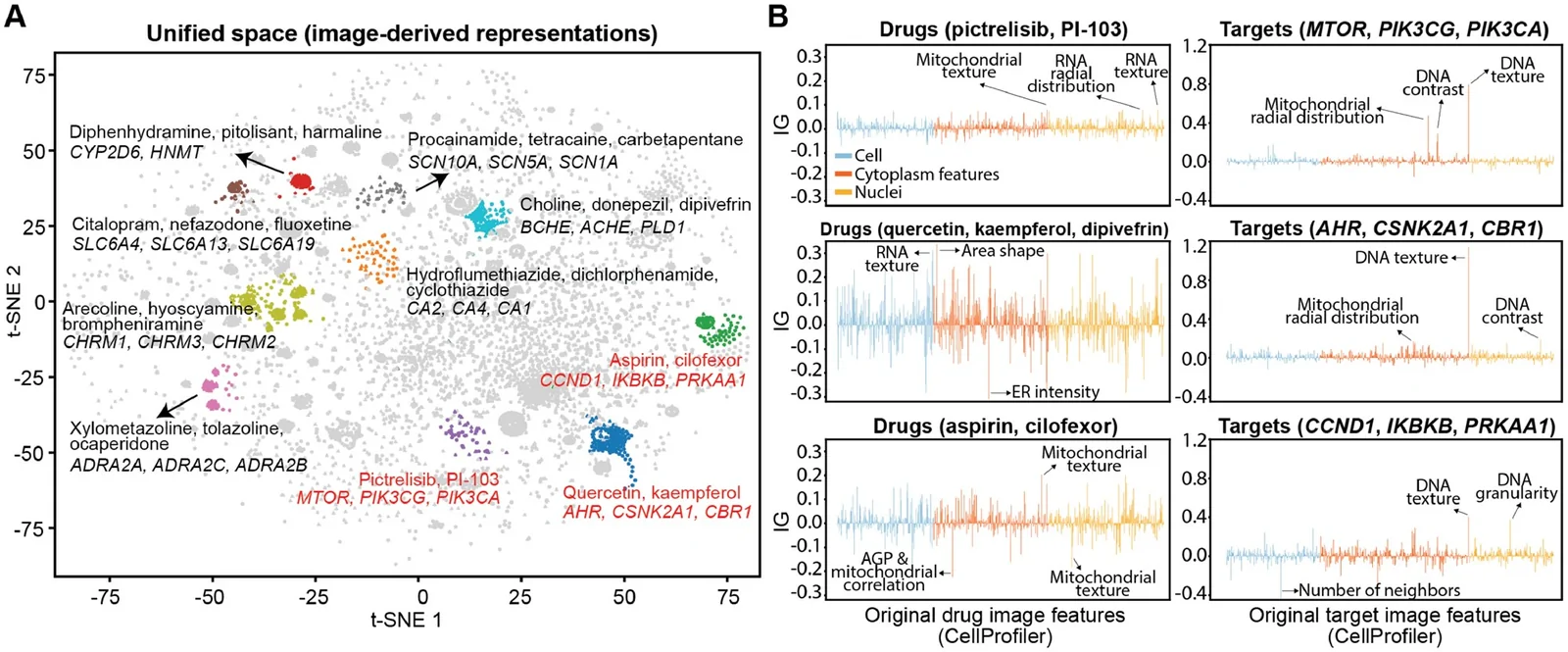
Interpretation of image-derived representations in the unified space. (A) t-SNE visualization with ten dense clusters annotated, with the three most frequent drugs and targets within each cluster labeled (or all entities shown when fewer than three are present). (B) Integrated gradients (IG) attribution profiles for representative drug-target pairs from distinct clusters, showing the top three CellProfiler features with the highest absolute IG scores.

To interpret the morphological features underlying the unified embedding space, we applied IG analysis to representative clusters identified by t-SNE projection (Fig. 4A), focusing on three mechanistically distinct groups: the PI3K-mTOR inhibitors (Pictilisib, PI-103), the flavonoids (quercetin, kaempferol), and the aspirin-cilofexor pair. The PI3K-mTOR cluster showed strong mitochondrial and RNA-related attributions at both drug and target levels (Fig. 4B), consistent with the pathway’s role in regulating cellular growth, metabolism, and organelle organization (Saxton and Sabatini 2017). The quercetin-kaempferol cluster was characterized by nuclear texture, cell shape, and ER intensity features, reflecting stress-associated changes in nuclear and organelle organization typical of flavonoid activity (Khan *et al.* 2024). In contrast, the aspirin-cilofexor cluster exhibited prominent attributions related to mitochondrial texture, actin-Golgi-plasma membrane correlations, and neighborhood features at the drug level, while DNA texture and granularity were most influential at the target level, consistent with shared involvement in cell cycle regulation, inflammation, and metabolism (Zhang *et al.* 2018, Younis *et al.* 2023). Together, these findings indicate that the unified embedding space provides interpretable evidence of biologically coherent drug-target representations.

## 4 Conclusion

In this study, we presented a unified multimodal framework for DTI prediction that integrates drug SMILES strings, protein sequences, and Cell Painting morphological profiles into a shared embedding space through a two-stage contrastive learning strategy. The learned representations demonstrate strong biological coherence, evidenced by improved separation between interacting and non-interacting pairs, robust cross-modal retrieval, and enhanced predictive accuracy on external benchmarks. Notably, the proposed contrastive learning framework alone outperforms existing structure-based methods, while incorporation of image-based cellular phenotypes provides additional complementary gains beyond molecular representations alone. IG analysis further confirms that the unified embeddings preserve pathway-specific morphological signatures, providing interpretable insights into drug mechanisms.

These results highlight the potential of multimodal representation learning to advance computational drug discovery by improving generalization to novel compounds and targets. Notably, we explored LoRA-based fine-tuning of ChemBERTa-2 and ESM-2 but found that frozen pretrained encoders yielded comparable performance, suggesting that the pretrained representations are sufficiently expressive for the contrastive learning objectives employed here. One limitation of this study is the smaller image-based DTI dataset, primarily due to limited availability of Cell Painting profiles. Future work may extend this framework to additional omics modalities and disease-specific contexts, as well as incorporate larger and more balanced phenotypic datasets, broadening its applicability in precision medicine.

## Data Availability

The drug-target interaction pairs used for training and evaluation were derived from the MOTIVE dataset (https://github.com/carpenter-singh-lab/2024_Arevalo_NeurIPS_MotiVE), retaining only direct binding interactions. Cell Painting morphological profiles for compounds and CRISPR knockouts were obtained from the JUMP Cell Painting Consortium dataset cpg0016 (https://github.com/jump-cellpainting/datasets), available through the Cell Painting Gallery. All source code and tutorials are freely available at https://github.com/YJRubyLai/Unified-DTI.
